# Screening for Neurocognitive Abilities Post-COVID (SNAP-COVID): Scale Development and Validation

**DOI:** 10.3390/medicina62061149

**Published:** 2026-06-12

**Authors:** Flora Nikolaou, Ioulia Solomou, Maria Loizidou, Panagiotis Papettas, Eleni Giorgoudi, Kalia Lofitou, Fofi Constantinidou

**Affiliations:** 1 Center for Applied Neuroscience, University of Cyprus, Nicosia 1678, Cyprus; nikolaou.flora@ucy.ac.cy (F.N.); isolom01@ucy.ac.cy (I.S.); loizidou.maria.3@ucy.ac.cy (M.L.); papettas.panayiotis@ucy.ac.cy (P.P.); giorgoudi.eleni@ucy.ac.cy (E.G.); lofitou.kalia@ucy.ac.cy (K.L.); 2 Department of Psychology, University of Cyprus, Nicosia 1678, Cyprus

**Keywords:** post-COVID condition, cognitive impairment, scale development, psychometric validation

## Abstract

*Background and Objectives*: The neurocognitive sequelae of COVID-19 have attracted attention as part of post-COVID condition (PCC), yet standardized tools for screening and quantifying PCC-related cognitive impairment remain scarce. The present study aimed to develop and validate the Screening for Neurocognitive Abilities Post-COVID (SNAP-COVID), a self-report questionnaire designed to capture current symptom burden and perceived changes in cognitive functioning relative to pre-COVID status in a Greek-speaking sample. *Materials and Methods*: Data collection occurred in three phases between August 2024 and February 2025. Dataset A (*N* = 27) was used for test–retest reliability. Dataset B (*N* = 300) was used for exploratory factor analysis (EFA), reliability testing, and convergent validity analyses with the Brain Fog Scale (BFS). Dataset C (*N* = 317) was used for independent validation through confirmatory factor analysis (CFA). *Results*: Initial EFA of the 30-item SNAP-COVID scale suggested a four-factor model, yet further item refinement yielded a robust three-factor, 24-item solution: (1) General Cognitive Functions (17 items, α = 0.948), (2) Sensory Hypersensitivity (4 items, α = 0.829), and (3) Language and Communication (3 items, α = 0.950). The total scale demonstrated excellent internal consistency (α = 0.95). Convergent validity was evident by significant correlations between SNAP impact scores and BFS scores (r = −0.442, *p* < 0.001). CFA confirmed the three-factor structure with acceptable fit indices (χ^2^(249) = 677.29, *p* < 0.001; CFI = 0.882; TLI = 0.869; RMSEA = 0.074; SRMR = 0.032). *Conclusions*: The SNAP-COVID scale is a reliable and valid instrument. Its multidimensional structure captures global and domain-specific difficulties, addressing a critical gap in post-infectious cognitive assessment.

## 1. Introduction

Disruptions caused by respiratory infections can affect multiple organ systems [1]. Respiratory infections can be acute or chronic, affecting the lungs and the general respiratory system [2]. Such infections may lead to primary symptoms which are caused directly by the infection (e.g., shortness of breath, coughing, wheezing) [3]. However, secondary symptoms, such as headaches, fatigue and cognitive dysfunction, may also arise, although not directly related to the primary infection per se [3,4].

Research has begun to unravel the underlying mechanisms through which respiratory infections can affect brain structure and function, informing our understanding of the pathways that contribute to the observed cognitive impairments. Chronic hypoxia and neuroinflammation, which are common in respiratory infections, can reduce oxygen supply to critical brain regions resulting in neuronal injury and measurable reductions in gray matter volume [5]. The hippocampus, known for its role in memory consolidation, is among the most vulnerable structures due to its high sensitivity to hypoxia [6,7]. Thus, findings suggest that complex interactions between systemic inflammation, oxygen deprivation, and neural network disruption, following chronic respiratory conditions, can contribute to cognitive decline.

The recent pandemic caused by SARS-CoV-2, more commonly referred to as COVID-19, has brought to the forefront the long-lasting effects of respiratory infections. Lung inflammation and hypoxia due to COVID-19 [8] may lead to chronic conditions such as post-COVID condition (PCC). PCC is defined by the persistence of symptoms more than three months after acute COVID-19 infection and is estimated to affect up to 20% of individuals infected with COVID-19 [9,10,11].

Ongoing secondary neurocognitive symptoms have also been reported in patients with PCC, through various pathways. By penetrating the blood–brain barrier, the virus may destroy multiple areas responsible for cognitive functions [12,13]. Further, it has been argued that neuronal retrograde transport through the olfactory nerve and bulb is associated with the frequent occurrence of anosmia among the affected individuals. Lastly, by triggering a strong neuroinflammatory response mediated by cytokine storms, microglial activation, and spike protein interaction, it may lead to prolonged immune activation and consequently brain tissue damage. Taken together, these mechanisms may contribute to the observed cognitive decline in patients recovering from acute COVID-19 [14], contributing to difficulties such as inattention and cognitive dysfunction [15,16].

Importantly, PCC is a highly heterogenous condition, with over 200 recognized symptoms that persist for more than three months [9], affecting all organ systems [17]. Thus, it becomes particularly difficult to determine whether symptoms are directly attributable to PCC or relate to other comorbid conditions, contributing to misdiagnosis or delayed care [18]. Emerging research indicates that shared pathological mechanisms, such as chronic hypoxia, systemic inflammation, and neuroinflammation, may underlie both PCC and other respiratory conditions, offering a potential explanation for overlapping symptoms, particularly in relation to cognitive dysfunction [19].

However, the lack of standardized criteria for diagnosing PCC-related cognitive impairment presents additional challenges for both clinicians and researchers [13,20]. Cognitive symptoms vary among individuals, increasing the risk of misdiagnosis or misattribution to unrelated conditions [21]. These challenges are further compounded by the influence of overlapping symptoms, such as chronic fatigue and depression, which can obscure or mimic cognitive deficits and confound assessment outcomes [22]. Researchers have attempted to develop specialized scales to measure these symptoms, such as the development of the Brain Fog Scale [16]. However, such tools rarely distinguish between symptoms that predate the COVID-19 infection and those that emerged because of it [23]. This differentiation is essential for accurate diagnosis and treatment planning yet is rarely addressed in current assessment protocols. Furthermore, limited awareness among clinicians means that cognitive dysfunction may be often overlooked as a key symptom of PCC, leading to underdiagnosis and inadequate treatment [24].

In response to these challenges, the present study aimed to develop and validate a self-report screening tool for assessing perceived neurocognitive symptoms associated with post-COVID condition in individuals with self-reported post-COVID cognitive complaints, as well as to identify how core cognitive abilities may be affected following COVID-19 infection. By addressing the current lack of standardized tools for evaluating cognitive symptoms in this population, the study sought to support more accurate diagnosis and facilitate timely interventions. Ultimately, this research contributes to the development of a structured, evidence-based approach for identifying and monitoring neurocognitive difficulties in individuals affected by PCC, enhancing both clinical practice and long-term patient care.

## 2. Materials and Methods

This research was part of a larger-scale study under the European Twinning initiative “Widening Participation & Strengthening the ERA”. The broader project, “Brain Research and Integrative Neuroscience Network for COVID-19 (BRAINN-COVID)”, is funded by the European Union’s Horizon Europe research and innovation program under grant agreement no. 101079001. The study was conducted in accordance with the Declaration of Helsinki and was approved by the Cyprus National Bioethics Committee (EEBK/EΠ/2023/40).

### 2.1. Participants and Data Collection Procedure

The study adhered to the Ten Steps in Scale Development and Reporting [25], following a structured, multi-phase approach to ensure the psychometric robustness of the newly developed questionnaire. The survey was hosted on the Research Electronic Data Capture (REDCap) platform [26], which offers secure and reliable tools for online data collection. All questionnaire fields were configured as mandatory within the survey platform; therefore, incomplete submissions were not permitted. Prior to analysis, records with incomplete data were excluded, and only participants with complete data relevant to each analysis were included in the corresponding datasets. No imputation procedures were applied for missing values.

Participants were recruited through a combination of mixed random and snowball sampling methods to ensure a diverse and adequate sample size, particularly with respect to varying COVID-19 experiences. The survey was disseminated via social media platforms, including targeted outreach to post-COVID condition (PCC) community groups, as well as through institutional and clinical mailing lists.

Eligibility criteria included being at least 18 years of age and fluent in the Greek language. No exclusion criteria were applied with respect to COVID-19 status, allowing inclusion of individuals with varying COVID-19 histories and symptom experiences. This approach was intended to capture a broad spectrum of perceived post-COVID cognitive symptom presentations and facilitate examination of symptom attribution relative to pre-COVID functioning.

Data collection was conducted in three phases. The first phase took place in August 2024 and included a cohort of 27 participants (Dataset A) who completed the scale twice within a span of two weeks, to assess test–retest reliability.

Dataset B was derived from the second phase, which was conducted in two consecutive recruitment rounds. In the first round (conducted between October 2024 and November 2024), 156 responses were collected. Given that the sample size at this stage was insufficient to support exploratory factor analysis (EFA), and in accordance with prior recommendations regarding factor stability and model fit [26], another data collection round was implemented between December 2024 and February 2025. During this round, an additional 144 responses were collected. These two data collection rounds were combined to form Dataset B (*N* = 300), which was used for EFA, internal consistency evaluation, and group comparisons.

**Figure 1:**
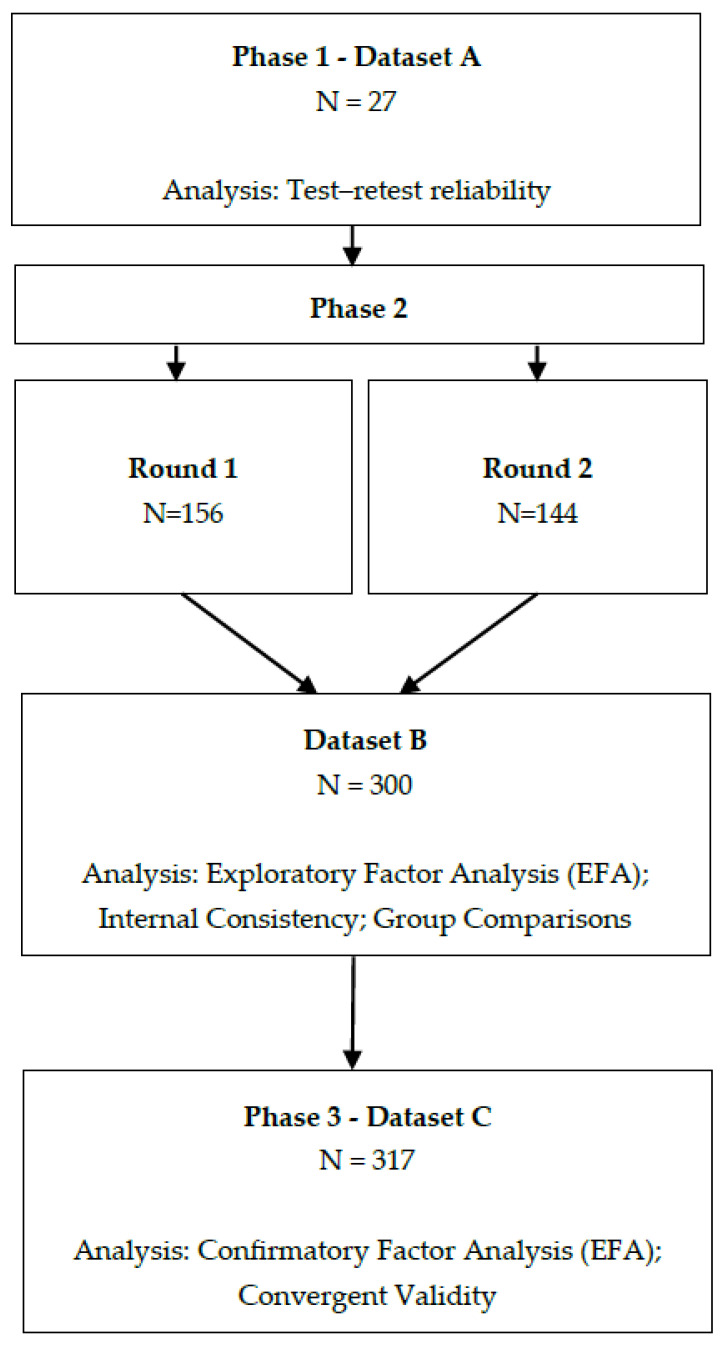
Overview of the three data collection phases.

To ensure the robustness of the final model and assess its generalizability, a third independent data collection phase was carried out between March 2025 and April 2025. This phase yielded an additional 317 responses (Dataset C), which were used exclusively for confirmatory factor analysis (CFA) and convergent validity assessment. Figure 1 provides an overview of the data collection procedure, and sample characteristics across the three datasets are presented in Table 1.

### 2.2. Scale Development Procedure and Other Instruments

#### 2.2.1. SNAP

The SNAP-COVID questionnaire was developed as the primary instrument for this study, with the objective of identifying and quantifying self-reported cognitive symptoms following COVID-19 infection. The scale was designed to capture both the intensity of specific cognitive symptoms experienced over the past two weeks and perceived changes in these symptoms relative to functioning prior COVID-19 infection. This allows for both the assessment of current symptoms and the impact of COVID-19 on cognitive functioning.

To guide the scale development process, we followed the Ten Steps in Scale Development and Reporting framework [27]. The process began with a comprehensive review of the literature on cognitive dysfunction associated with PCC and other post-infection diseases which informed the conceptualization of seven key cognitive domains: attention, processing speed, language, memory, mental fatigue, executive functions, and visuospatial skills. These domains served as the initial theoretical framework for item generation and were subsequently evaluated and refined empirically through exploratory factor analysis.

Based on these domains, a detailed set of items were prepared. Each item included a two-part response format: (1) symptom severity during the past two weeks, rated on a 4-point Likert scale (1 = Never or Rarely, 2 = Sometimes, 3 = Quite Often, 4 = Very Often or Always), and (2) perceived change in the symptom following COVID-19 infection, rated as Improved (1), No Change (0), or Worsened/New Symptom (−1). For each item, an impact score was calculated by multiplying the severity score by the perceived change score. This resulted in item-level impact scores ranging from −4 to +4, with more negative values indicating greater symptom worsening attributed to COVID-19.

An initial pool of 28 items was developed to capture observable behaviors representing the identified cognitive domains. Item generation was guided by the conceptual definitions of each domain identified through the literature review. Specifically, items targeting attention focused on distractibility and sustained concentration difficulties; executive functioning items addressed planning, organization, and multitasking; memory items targeted forgetting and recall difficulties; language items focused on word-finding and verbal expression; processing speed items reflected slowed thinking and delayed responses; mental fatigue items addressed cognitive exhaustion; and visuospatial items targeted spatial awareness and visual processing difficulties.

The preliminary item pool was reviewed by five experts with clinical and research experience in post-COVID condition, neuropsychology, cognitive rehabilitation, and psychological assessment. Experts evaluated the relevance, clarity, comprehensibility, and clinical appropriateness of each item and provided qualitative feedback regarding wording, redundancy, and domain representation, which informed iterative item revision and refinement. In addition, feedback was solicited from individuals with lived experience of post-COVID cognitive difficulties to ensure linguistic clarity and ecological relevance of the items. Although a formal Content Validity Index (CVI) was not calculated, iterative revisions were made based on expert and participant feedback prior to finalization of the scale.

Following this review and iterative revisions based on expert and patient feedback, two additional items were added, resulting in a final scale comprising 30 items. This version ensured broader construct representation and improved clarity across items, while retaining a practical format for clinical and community-based use.

#### 2.2.2. Brain Fog Questionnaire

To further assess convergent validity, participants in the fourth phase of data collection also completed the Brain Fog Scale (BFS). The BFS is a 23-item self-report instrument designed to measure the severity and frequency of symptoms commonly associated with brain fog, such as concentration difficulties, memory lapses, mental fatigue, and slowed thinking. Each item is rated on a 5-point Likert scale (0 = Never, 1 = Rarely, 2 = Occasionally, 3 = A lot of the time, 4 = Nearly all the time) reflecting the extent to which the respondent has experienced each symptom within a specified timeframe.

For the purposes of this study, the Greek version of the BFS was administered, after being linguistically and culturally adapted for the Greek-speaking population. This version retains the original structure and psychometric properties of the instrument while ensuring linguistic appropriateness and conceptual equivalence.

#### 2.2.3. COVID-19 Information

Participants indicated their COVID-19 infection history using a single binary-response item: “Have you ever had a positive COVID-19 test result?” (Yes/No). Participants who responded positively were then asked to indicate the number of times they had tested positive for COVID-19. Responses included once, twice, three times, four times, five times and above.

To assess PCC, participants were first presented with the World Health Organization’s clinical case definition of long COVID: “Post COVID-19 condition occurs in individuals with a history of probable or confirmed SARS-CoV-2 infection, usually three months from the onset of COVID-19, with symptoms that last for at least two months and cannot be explained by an alternative diagnosis” [9]. Afterwards participants were presented the following question: “Based on the above definition, have you ever received a Long COVID diagnosis?” (Yes/No).

Participants who responded negatively were subsequently asked, “Based on the above definition, do you believe you should have received a Long COVID diagnosis?” (Yes/No). This additional item aimed to capture subjective symptom attribution in the absence of a formal diagnosis.

### 2.3. Statistical Analysis Plan

All statistical analyses were conducted using IBM SPSS Statistics for Windows, Version 28.0 (IBM Corp., Armonk, NY, USA), except for confirmatory factor analysis (CFA), which was conducted using IBM SPSS Amos Version 29.0 (IBM Corp., Armonk, NY, USA).

Internal consistency of the original 30-item version of the SNAP questionnaire was evaluated using Cronbach’s alpha, on Dataset A. Test–retest reliability was assessed with Dataset A (*N* = 2), where participants completed the measure twice, approximately two weeks apart. Pearson correlations and intraclass correlation coefficients (ICCs) were calculated to evaluate score stability over time.

Exploratory factor analysis (EFA) using principal axis factoring with oblimin rotation was then performed on Dataset B (*N* = 300), to examine the underlying structure of the original 30-item scale (SNAP). Factor retention was based on eigenvalues greater than 1.0, scree plot inspection, and theoretical interpretability.

Following factor refinement, impact scores were calculated to estimate the degree to which each reported symptom was attributed to COVID-19 (Section 2.2.1). Impact scores were computed by multiplying the severity rating of each item by its respective change rating (post-COVID). Subscale-level and total impact scores were computed by averaging the corresponding item-level impact values. Descriptive statistics were calculated, and scores were categorized into interpretive levels (e.g., high, moderate, low, improved) based on their distribution.

To assess convergent validity, Pearson correlations were computed between the SNAP impact scores and the total score from a validated Brain Fog Scale, using complete records of Dataset B (*N* = 274). Records with incomplete data on either measure were excluded from this analysis. It was hypothesized that higher levels of COVID-related cognitive burden would be associated with greater levels of self-reported brain fog.

Finally, a confirmatory factor analysis (CFA) was conducted using Dataset C as an independent dataset (*N* = 317) to test the stability of the three-factor structure identified via EFA. Model fit was evaluated using the following fit indices: Comparative Fit Index (CFI), Tucker–Lewis Index (TLI), Root Mean Square Error of Approximation (RMSEA), and Standardized Root Mean Square Residual (SRMR). Standardized factor loadings are reported in the following sections.

## 3. Results

### 3.1. Dataset A: Internal Consistency and Test–Retest Reliability

The internal consistency and temporal stability of the SNAP questionnaire were evaluated using participants from Dataset A (*N* = 27) who completed the instrument at two timepoints approximately two weeks apart.

#### 3.1.1. Internal Consistency

Cronbach’s alpha coefficients were calculated for the total 30-item scale and for each of the initially theorized subscales. The overall internal consistency of the full scale was excellent (α = 0.896), indicating high internal reliability. Subscale reliability varied, with language (α = 0.725) and sensory-related (α = 0.739) subscales demonstrating acceptable internal consistency. The subscales of memory (α = 0.613), attention (α = 0.587), and mental fatigue (α = 0.604) showed questionable reliability. Processing speed (α = 0.574) and executive functions (α = 0.509) were in the poor range, while visuospatial skills (α = 0.292) were unacceptable. These findings informed the subsequent refinement of the scale structure.

#### 3.1.2. Test–Retest Reliability

Test–retest reliability was assessed using the intraclass correlation coefficient (ICC) based on a two-way mixed effects model. Results revealed average-measures ICCs of 0.955 (95% CI [0.924, 0.977], *p* < 0.001), indicating the excellent test–retest reliability of the overall scale.

### 3.2. Dataset B: Exploratory Factor Analysis

An exploratory factor analysis (EFA) was conducted to examine the latent structure of the 30-item SNAP questionnaire and to guide refinement of the scale. Data were drawn from a sample of 300 participants (Dataset B).

#### 3.2.1. Initial EFA (30 Items)

Data showed excellent sampling adequacy (Kaiser–Meyer–Olkin = 0.951) and sphericity (Bartlett’s test, *χ*^2^(435) = 5226.15, *p* < 0.001). EFA revealed a four-factor solution which explained 53.27% of the total variance. These factors showed acceptable separation in the pattern matrix. However, upon closer inspection, several items showed low communalities, poor loadings, or weak internal consistency within their subscale groupings.

Cronbach’s alpha values for the initial four factors were as follows: Factor 1 (general cognitive functions): α = 0.934, Factor 2 (sensory hypersensitivity and cognitive overload): α = 0.819, Factor 3 (language and communication): α = 0.836, and Factor 4: α = 0.651. Factor 4, which consisted of only three items (snap_15, snap_23, and snap_25; see Table 2 for details), exhibited limited conceptual cohesion and relatively modest loadings (0.405–0.598). Eigenvalues for the items comprising Factor 4 were 3.00, 2.99, and 2.86. These were considerably lower than the eigenvalues for Factor 1, which had an eigenvalue of 21.36, suggesting significantly weaker explanatory power. Consequently, it was deemed more appropriate to remove these three items from the scale.

Additionally, three more items (snap_3, snap_4, and snap_21; see Table 2 for details) were excluded due to low primary factor loadings below 0.40 (ranging from 0.331 to 0.379) and lack of theoretical clarity within the factor structure. These decisions were made to improve overall model fit, simplify interpretation, and increase the internal reliability of the retained factors.

#### 3.2.2. Refined EFA (24-Item Version)

A second EFA was conducted on the remaining 24 items. Sampling adequacy remained excellent (KMO = 0.952) and Bartlett’s test of sphericity remained highly significant; *χ*^2^(276) = 4387.26, *p* < 0.001. The refined analysis yielded a robust three-factor solution, explaining 55.43% of the variance. This empirically derived structure differed from the initially theorized conceptual domains, indicating substantial overlap among several cognitive symptom categories in the studied population. All retained items demonstrated primary loadings greater than 0.40 with minimal cross-loadings.

Following careful assessment of scale items in each factor, Factor 1 was labeled “General Cognitive Functions” (17 items) with Cronbach’s α = 0.948, Factor 2—“Sensory Hypersensitivity” (4 items) with α = 0.829, and Factor 3—“Language and Communication” (3 items) with α = 0.950. Correlations between factors ranged from 0.52 to 0.70, indicating moderate to strong relationships among the three extracted factors. The total 24-item SNAP scale demonstrated excellent internal consistency, with Cronbach’s α = 0.95. Standardized factor loadings for the three extracted factors are presented in Table 3.

#### 3.2.3. Impact Score Results

Following refinement of the SNAP questionnaire to a 24-item structure, impact scores were calculated to quantify the degree to which symptoms were attributed to COVID-19 among individuals with PCC and those without a diagnosis. These scores were based on responses in symptom severity and the reported change in that symptom following COVID-19 infection. Subscale and total impact scores were subsequently calculated by averaging the corresponding item-level impact scores, with more negative values reflecting greater perceived COVID-related symptom burden.

Descriptive analyses of the total impact score revealed a slightly left-skewed distribution, with a mean of −0.49 (SD = 0.99). A large portion of participants scored at or near zero, suggesting either minimal symptoms or symptoms unrelated to COVID-19. However, a notable portion of the sample demonstrated negative impact scores, reflecting a moderate to high burden of post-COVID cognitive symptoms.

**Figure 2:**
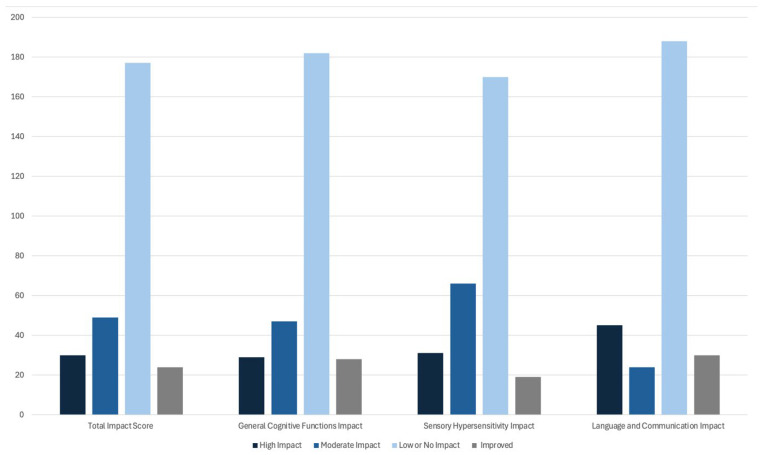
Distribution of Participants Across SNAP-COVID Impact Score Categories for Each Validated Factor. Note. The figure displays the frequency of participants classified within each SNAP-COVID impact score category (high impact, moderate impact, low or no impact, and improvement) across the validated subscales and total impact score. Categories were derived from averaged impact scores as described in the statistical analysis section. High-impact subscale scores are represented in dark blue, moderate-impact scores in blue, low or no-impact scores in light blue and improvement scores in gray.

Subscale-level impact scores were computed by averaging the item-level impact scores within each of the three validated factors. The general cognitive functions subscale (17 items) had a mean impact score of −0.45 (SD = 0.997), the sensory hypersensitivity subscale (4 items) had a mean score of −0.53 (SD = 1.06), and the language and communication subscale (3 items) had a mean score of −0.52 (SD = 1.24). These findings indicate that, on average, participants reported minimal worsening of symptoms across all three domains (Figure 2).

For descriptive and interpretive purposes, total and subscale impact scores were categorized into four levels: high impact (≤−2.0), moderate impact (−1.99 to −0.75), low or no impact (−0.74 to 0), and improved (>0). These cut-off points were derived empirically based on the observed distribution of scores within the study sample and were informed by approximately one standard deviation around the mean impact score. The categories were intended to provide clinically interpretable groupings for descriptive analyses rather than diagnostic thresholds and should therefore be interpreted cautiously pending further validation in independent samples [28].

#### 3.2.4. Convergent Validity

To examine convergent validity, SNAP impact scores were correlated with the Brain Fog Scale total scores. Pearson correlation analyses demonstrated statistically significant, negative associations between the total SNAP impact score and brain fog (*r* = −0.442, *p* < 0.001), indicating that participants who reported more COVID-related cognitive difficulties also reported higher levels of brain fog. This pattern was consistent across all three subscales. The general cognitive functions subscale showed a correlation of *r* = −0.424 (*p* < 0.001), the sensory hypersensitivity subscale correlated at *r* = −0.347 (*p* < 0.001), and the language and communication subscale correlated at *r* = −0.399 (*p* < 0.001). Correlations between the SNAP impact scores and the brain fog total score are summarized in Table 4.

### 3.3. Dataset C: Confirmatory Factor Analysis

A confirmatory factor analysis (CFA) was conducted to examine the proposed three-factor structure of the scale, composed of general cognitive functions, sensory hyperresponsivity, and language and communication.

#### 3.3.1. CFA

The model demonstrated an overall adequate fit to the data. Although the Comparative Fit Index (CFI = 0.882) and Tucker–Lewis Index (TLI = 0.869) fell slightly below the conventional 0.90 threshold for acceptable fit, the RMSEA (0.074) and SRMR (0.032) values supported an acceptable approximation of model fit. Given the exploratory nature of the scale development process and the strong theoretical coherence of the retained factors, the three-factor structure was retained for further validation. All standardized factor loadings were statistically significant (*p* < 0.001), ranging from 0.345 to 0.794. The majority of items had loadings above 0.50, indicating adequate convergent validity within each factor. The three latent constructs were significantly and positively correlated, supporting the theoretical relatedness of the domains.

#### 3.3.2. Between-Group Comparisons

A one-way multivariate analysis of variance (MANOVA) was conducted to examine the effect of COVID-19 recovery status (1 = not recovered, 2 = recovered) on three dependent variables: general cognitive functions impact, sensory hyperresponsivity impact, and language and communication impact. The multivariate test revealed a statistically significant effect of COVID-19 recovery status on the combined dependent variables, Pillai’s Trace = 0.070, *F*(3, 254) = 4.771, *p* < 0.001, partial *η*^2^ = 0.070, indicating a small to moderate multivariate effect.

Follow-up univariate ANOVAs revealed significant group differences for three subscales. Participants who had not recovered from COVID-19 reported significantly higher language/communication difficulties, *F*(1, 256) = 15.997, *p* < 0.001, partial *η*^2^ = 0.059; greater general cognitive difficulties, *F*(1, 256) = 5.526, *p* = 0.019, partial *η*^2^ = 0.021. However, the difference in sensory hyperresponsivity impact between the groups did not reach statistical significance; *F*(1, 256) = 1.746, *p* = 0.188, partial *η*^2^ = 0.007.

To complement the subscale-level analyses, an additional univariate ANOVA was conducted on the total SNAP impact score. Results showed that participants who had not recovered from COVID-19 also reported significantly higher overall impact, F(1, 256) = 5.513, *p* = 0.020, partial *η*^2^ = 0.021. For interpretive clarity, the analyses presented in Table 5 were conducted using raw summed SNAP impact scores rather than averaged impact scores reported in previous analyses. Group comparisons between COVID-recovered and non-recovered participants are presented in Table 5.

## 4. Discussion

This study aimed to develop and validate the SNAP-COVID questionnaire, a screening tool designed to assess cognitive symptoms related to post-COVID condition (PCC). The findings support the SNAP-COVID as a reliable psychometric instrument that captures a wide range of neurocognitive symptoms attributed to infection-related conditions. The scale demonstrated excellent internal consistency, strong test–retest reliability, and a clear three-factor structure encompassing general cognitive functions, sensory hypersensitivity, and language and communication. Furthermore, its convergent validity with an established brain fog questionnaire underscores its clinical and research relevance in evaluating post-infectious cognitive sequelae. Importantly, the SNAP-COVID was developed as a self-report screening instrument for perceived post-COVID cognitive symptoms rather than as a diagnostic tool for clinically confirmed post-COVID condition. The proposed three-factor structure was confirmed via confirmatory factor analysis (CFA), which demonstrated acceptable model fit and all standardized factor loadings were statistically significant. Most item loadings exceeded 0.50, suggesting strong convergent validity. The three latent constructs were also positively correlated, supporting the theoretical interrelatedness of the symptom domains.

The SNAP-COVID’s scale convergent validity was supported through significant correlations with the Brain Fog Scale (BFS). Moderate-to-strong negative correlations were observed between SNAP impact scores and BFS total scores across all three domains and the total scale. This indicates that individuals who reported more COVID-related cognitive difficulties also reported higher levels of brain fog, supporting the tool’s ability to detect real-world cognitive symptoms as experienced by patients. These findings support the hypothesis that brain fog, characterized by impaired attention, memory issues, and slowed information processing, is a core feature of post-infectious cognitive syndromes, including PCC [29]. The SNAP-COVID’s domain-specific design builds upon existing tools by differentiating symptom clusters, making it particularly useful for tracking differential responses to treatment or rehabilitation.

The impact score results aligned with previous findings highlighting persistent cognitive difficulties in individuals with PCC, particularly in domains such as attention, executive functioning, and memory [30,31]. Difficulties in language and communication also emerged clearly, consistent with prior studies reporting slowed verbal processing and word-finding issues in PCC cohorts [32]. Sensory-related symptoms were also reported, which aligns with emerging evidence regarding sensory overload and environmental intolerance, as common yet underrecognized features of post-viral neurocognitive impairment [33,34]. However, there was no difference in the reporting of sensory symptoms between participants who self-identified as recovered and those who reported PCC-related difficulties at the time of participation. This suggests that while sensory symptoms are relevant and present across our cohort, they may be present even after PCC has resolved, potentially reflecting more persistent or baseline-level disruptions that extend beyond subjective recovery [34]. Significant group differences emerged for general cognitive difficulties, language/communication impact, and total impact scores, with non-recovered individuals reporting greater burden. It should also be pointed out that symptoms measured by SNAP are not unique to SARS-CoV-2; similar post-infectious syndromes following other viral illnesses, such as Epstein–Barr virus and influenza, have also been associated with cognitive dysfunction, fatigue, and sensory disturbances [35,36].

The three-factor structure identified through exploratory factor analysis (general cognitive functions, sensory hypersensitivity, language and communication) is consistent with existing research suggesting that infection-related cognitive changes are multifaceted and not limited to a single domain [32,37]. This multidimensional structure allows the SNAP-COVID to provide a nuanced symptom profile, enhancing its utility for both clinical triage and longitudinal monitoring. Importantly, the tool’s focus on perceived symptom changes compared to pre-infection functioning provides a methodologically sound approach to isolating infection-related changes from pre-existing conditions.

### 4.1. Clinical and Research Implications

Given the prevalence of infection-related cognitive symptoms, the development of a standardized and culturally appropriate cognitive screening tool is critical. The SNAP-COVID provides a structured approach for identifying individuals who may benefit from further neuropsychological evaluation or rehabilitative support. Its two-part item design assessing both symptom severity and perceived post-infection change uniquely positions it to distinguish new cognitive deficits from longstanding or unrelated complaints.

In clinical practice, SNAP-COVID may serve as a preliminary self-report screening tool in community, primary care, and post-infection settings to help identify individuals who may require further clinical or neuropsychological evaluation. In research, the scale enables stratification of participants in clinical trials or observational studies and supports hypothesis-driven exploration of mechanisms underlying infection-induced cognitive changes, such as neuroinflammation, hypoxia, and blood–brain barrier disruption [38]. Future studies integrating objective cognitive performance measures alongside SNAP-COVID scores will be particularly important for establishing the scale’s clinical sensitivity and ecological validity.

### 4.2. Limitations and Future Directions

Despite the added value of this study and the SNAP-COVID tool, a few limitations must be acknowledged. First, although the sample size was adequate for scale validation, the recruitment strategy relied primarily on online dissemination through social media, post-COVID community groups, institutional channels, and clinical mailing lists, which may have introduced selection bias toward individuals with greater symptom awareness or concern regarding post-COVID symptoms. In addition, the sample was predominantly female, relatively young, and highly educated, which may limit the generalizability of the findings to broader and more clinically diverse populations. In addition, the sample used for test–retest reliability analyses was relatively small and therefore the stability estimates should be interpreted with some caution until being replicated in larger independent samples. Future studies should aim to replicate these findings in more diverse and clinically verified samples, including pediatric and geriatric populations. Additionally, post-COVID condition status was based primarily on self-report and perceived symptom attribution rather than formal clinical diagnosis, which may have introduced heterogeneity within the sample. Also, although the confirmatory factor analysis supported the proposed three-factor structure, some fit indices (CFI and TLI) fell slightly below conventional thresholds, suggesting that further refinement and replication of the model in independent samples is warranted. Second, the study relied exclusively on self-report measures and did not include objective neurocognitive assessments or discriminant validity testing using theoretically unrelated constructs. While self-report instruments are appropriate for initial symptom screening, future studies should incorporate standardized neuropsychological measures, neuroimaging findings, and discriminant validity analyses to further establish the specificity and construct validity of the SNAP-COVID scale [31,38]. Although the impact scoring approach was designed to capture symptom worsening relative to pre-COVID functioning, the use of negative values to indicate greater symptom burden may be less intuitive for clinical interpretation and may require further refinement in future applications. Third, the cross-sectional design precludes causal inferences regarding the evolution or persistence of symptoms. Longitudinal studies are needed to assess the SNAP-COVID’s sensitivity to change and its utility in monitoring recovery or treatment response over time. Fourth, the study did not test for comorbid conditions that could influence symptom perception such as depression, anxiety and post-traumatic disorder. Future studies should also investigate how increase in psychological symptoms may influence SNAP scores. As the scale was developed and validated in a Greek-speaking sample, its cross-cultural applicability requires further investigation. Translations and validations in other languages and healthcare systems are essential to ensure the scale’s generalizability and global utility. Finally, while developed in the context of COVID-19, the SNAP-COVID’s conceptual framework and item structure are grounded in broader principles of post-infectious neurocognitive impairment. Therefore, its potential extends beyond PCC. Given that cognitive symptoms are also prevalent in other infection-triggered syndromes [39] future validation studies could explore the SNAP-COVID’s adaptability and diagnostic utility across diverse infectious contexts.

## 5. Conclusions

The SNAP-COVID questionnaire is a reliable and valid instrument for assessing neurocognitive symptoms attributed to COVID-19 and other infections. Its multidimensional structure, robust psychometric properties, and domain-specific focus fill a critical gap in the assessment of post-infectious neurocognitive sequelae. By supporting early self-reported symptom identification and facilitating future research efforts, the SNAP-COVID represents an important step toward improving the assessment of neurocognitive dysfunction following infection.

## Figures and Tables

**Table 1:** Sample Description and COVID-19 Infection History Across Datasets.

	Dataset A (*N* = 27)	Dataset B (*N* = 300)	Dataset C (*N* = 317)
Gender			
Female	19 (70.4%)	241 (80.3%)	236 (74.4%)
Male	8 (29.6%)	59 (19.7%)	84 (26.5%)
Age			
18–29	12 (44.4%)	162 (54.0%)	192 (60.6%)
30–39	12 (44.4%)	55 (18.3%)	32 (10.1%)
40–49	1 (3.7%)	34 (11.3%)	34 (10.7%)
50–59	2 (7.4%)	32 (10.7%)	40 (12.6%)
60–69	0	15 (5.0%)	19 (6.0%)
70–79	0	2 (0.7%)	0
Education			
No formal education	0	2 (0.7%)	0
Primary school	0	0	2 (0.6%)
Middle school	0	0	2 (0.6%)
High school	3 (11.1%)	108 (36.0%)	113 (35.6%)
Diploma	2 (7.4%)	18 (6.0%)	26 (8.2%)
Bachelor’s degree	7 (25.9%)	88 (29.3%)	82 (25.9%)
Master’s degree	11 (40.7%)	65 (21.7%)	71 (22.4%)
Doctorate degree	4 (14.8%)	17 (5.7%)	20 (6.3%)
Other	0	2 (0.7%)	1 (0.3%)
Occupation			
Full-time employment	22 (81.5%)	135 (45.0%)	120 (37.9%)
Part-time employment	0	19 (6.3%)	36 (11.4%)
Retired	0	10 (3.3%)	8 (2.5%)
Student	4 (14.8%)	114 (38.0%)	136 (42.9%)
Unemployed	0	10 (3.3%)	2 (0.6%)
Not working	1 (3.7%)	10 (3.3%)	10 (3.2%)
Other	0	2 (0.7%)	5 (1.6%)
COVID-19 infection history			
No	1 (3.7%)	18 (6.0%)	56 (17.7%)
Yes	26 (96.3%)	282 (94.0%)	261 (82.3%)
Number of COVID-19 infections			
Once	10 (37.0%)	101 (33.7%)	117 (36.9%)
Twice	6 (22.2%)	104 (34.7%)	92 (29.0%)
Three times	7 (25.9%)	63 (21.0%)	43 (13.6%)
Four times	2 (7.4%)	11 (3.7%)	5 (1.6%)
Five or more times	1 (3.7%)	3 (1.0%)	1 (0.3%)

**Table 2:** Excluded SNAP-COVID Items.

Item No.	Item Description (Shortened/Paraphrased)
snap_3	Suddenly forget what I was saying or thinking.
snap_4	Often forget to finish daily tasks.
snap_15	Misjudge or overlook objects around me.
snap_21	Occasionally experience blurry vision.
snap_23	Misplace items like keys or glasses in odd places (e.g., fridge).
snap_25	Difficulty judging distances or depth.

Note. Item No refers to the number of the scale item in the SNAP-COVID scale.

**Table 3:** Standardized factor loadings for the three factors of Screening for Neurocognitive Abilities Post-COVID (SNAP-COVID).

Item No.	Item Description (Shortened/Paraphrased)	F1	F2	F3
snap_1	Trouble concentrating	0.703		
snap_2	Difficulty multitasking	0.564		
snap_5	Trouble finding words			0.797
snap_6	Forgetting recent events	0.7		
snap_7	Losing focus in conversations	0.487		
snap_8	Tinnitus interferes with activities		0.429	
snap_9	Overwhelmed by noise		0.698	
snap_10	Easily distracted	0.731		
snap_11	Trouble following instructions	0.652		
snap_12	Misplacing items	0.641		
snap_13	Difficulty switching tasks	0.473		
snap_14	Slower thinking than before	0.465		
snap_16	Mental fatigue from bright lights/sounds		0.777	
snap_17	Difficulty tolerating sounds		0.679	
snap_18	Repeating self in speech	0.682		
snap_19	Memory issues for names/conversations	0.785		
snap_20	Losing track of time	0.578		
snap_22	Delays in responding	0.613		
snap_24	Trouble planning ahead	0.552		
snap_26	Trouble solving problems	0.798		
snap_27	Mental disorganization	0.793		
snap_28	Impaired attention in conversations	0.545		
snap_29	Slowed verbal communication			0.649
snap_30	Difficulty articulating ideas			0.603

Note. F1 = general cognitive functions; F2 = sensory hypersensitivity; F3 = language and communication. All factor loadings are statistically significant at *p* < 0.05.

**Table 4:** Pearson Correlations Between SNAP Impact Scores and Brain Fog Total Score.

Measure	*Μ*	SD	1.	2.	3.	4.
1. Total SNAP Impact	−0.48	0.98				
2. General Cognitive Functions Impact	−0.45	0.99	0.926 **			
3. Sensory Hypersensitivity Impact	−0.52	1.05	0.85 **	0.730 **		
4. Language and Communication Impact	−0.52	1.24	0.88 **	0.744 **	0.559 **	
5. Brain Fog Total Score	38.14	19.31	−0.442 **	−042 **	−0.347 **	−0.399 **

Note. Higher impact scores reflect greater COVID-related symptom burden (more negative = more severe); ** *p* < 0.001 (2-tailed) for all correlations.

**Table 5:** Group Comparisons Using Raw SNAP Impact Scores.

	COVID-Non-Recovered Mean (*SD*)		COVID-Recovered Mean (*SD*)		*F*	*p*	Partial *η*^2^
Total SNAP Impact	10.26	18.73	5.35	12.97	5.513	0.020	0.021
General Cognitive Functions Impact	7.11	13.89	3.49	9.50	5.526	0.019	0.021
Sensory Hypersensitivity Impact	1.55	3.08	1.03	2.63	1.746	0.188	0.007
Language and Communication Impact	5.68	1.87	4.61	1.88	15.997	<0.001	0.059

## Data Availability

The data supporting the findings of this study are publicly available in a recognized research data repository at Zenodo: https://doi.org/10.5281/zenodo.18255932 (accessed on 21 May 2026).
